# Single-cell analysis reveals the potential mechanisms of pyrotinib resistance in non-small cell lung cancer

**DOI:** 10.1038/s41392-022-01226-1

**Published:** 2023-01-13

**Authors:** Xinfeng Wang, Yuan Li, Runsen Jin, Sufei Zheng, Yuejun Luo, Peng Wu, Zhanyu Wang, Yuxin Yao, Nan Sun, Jie He

**Affiliations:** 1 Department of Thoracic Surgery, National Cancer Center/National Clinical Research Center for Cancer/Cancer Hospital, Chinese Academy of Medical Sciences and Peking Union Medical College, Beijing, 100021 China; 2 Department of Oncology, Renmin Hospital of Wuhan University, Wuhan, 430060 China; 3 Department of Thoracic Surgery, Ruijin Hospital, Shanghai Jiao Tong University School of Medicine, Shanghai, 200025 China

**Keywords:** Lung cancer, Cancer genomics

**Dear Editor**,

Lung cancer still remains the leading cause of cancer death worldwide, 80–90% of which are non-small cell lung cancer (NSCLC). In 2–3% of NSCLC patients, mutations in human epidermal growth factor receptor 2 (HER2) have been identified as oncogenic drivers. However, HER2 has been poorly reported as a therapeutic target for advanced NSCLC despite of the outstanding improvements in breast cancer receiving HER2-targeting regimens. Recently, a novel oral, irreversible pan-HER tyrosine kinase inhibitor (TKI) against EGFR and HER2, pyrotinib, has shown antitumor effects on HER2-mutant NSCLC.^1,2^ Moreover, an in vitro study found acquired resistance after continuous exposure to pyrotinib,^3^ indicating that drug resistance may also limit long-term outcomes of pyrotinib treatment. Therefore, understanding the mechanisms of pyrotinib resistance is of great importance for its clinical application.

Although several putative resistance mechanisms have been identified based on bulk sequencing, these conventional strategies showed limited capacity of detecting clonal evolutions during therapies. Nowadays, single-cell RNA-sequencing (scRNA-seq) has emerged as a promising approach to profiling the transcriptomic characteristics of abundant individual cells, allowing us to resolve intratumor heterogeneity, understand tumor microenvironment, and identify rare populations. Also, scRNA-seq has been conducted to profile the dynamics of transcriptomic features during anticancer therapies and reveal the drug resistance mechanisms in cancers. Here in our current study, we utilized scRNA-seq to explore the particular alterations in transcriptomics and predictive functional pathways of NSCLC cells in response to pyrotinib, unraveling the underlying resistance mechanisms.

Our preliminary experiments revealed that H358 possessed relatively higher expression of HER2 and were more sensitive to pyrotinib than other NSCLC cell lines (Supplementary Fig. S1). As shown in Fig. 1a, we treated H358 cell line with pyrotinib at doses of 100 nM, 200 nM, 500 nM, and 1000 nM every four days, respectively. Then, the untreated cells (P0) as well as longitudinal samples after each administration (P4, P8, P12, P16) were harvested for further analysis. We profiled the single-cell transcriptomes of an average of ~8000 cells from each sample with an average of 4736 genes and 90 thousand reads detected every cell. After quality control, 8507, 7684, 7715, 9367, and 8531 cells in each sample respectively were included. All the cells were divided into 16 clusters, shown by Uniform Manifold Approximation and Projection (UMAP) analysis for dimension reduction (Fig. 1b). When these clusters of cells were mapped to samples, we found that the untreated cells had the different gene expression pattern distinct from other samples (Fig. 1c). Then, the transcriptomic evolution of cell clusters during the increase of drug doses over time was revealed by UMAP plots separated by different samples (Fig. 1d) and a Sankey diagram (Supplementary Fig. S2a). Further detailed mapping relationships between clusters and samples were demonstrated by cell distributions separately in different samples or clusters (Supplementary Fig. S2b–g). Intriguingly, an isolated subpopulation, Cluster 15, was identified in the sample P16 (Fig. 1b–d). These data together showed that the untreated cells (P0) and the persistent cells (P16) had distinct and stable expression patterns whereas the samples between them shared similar transcriptomic profiles and were undergoing transitional states.

**Fig. 1:**
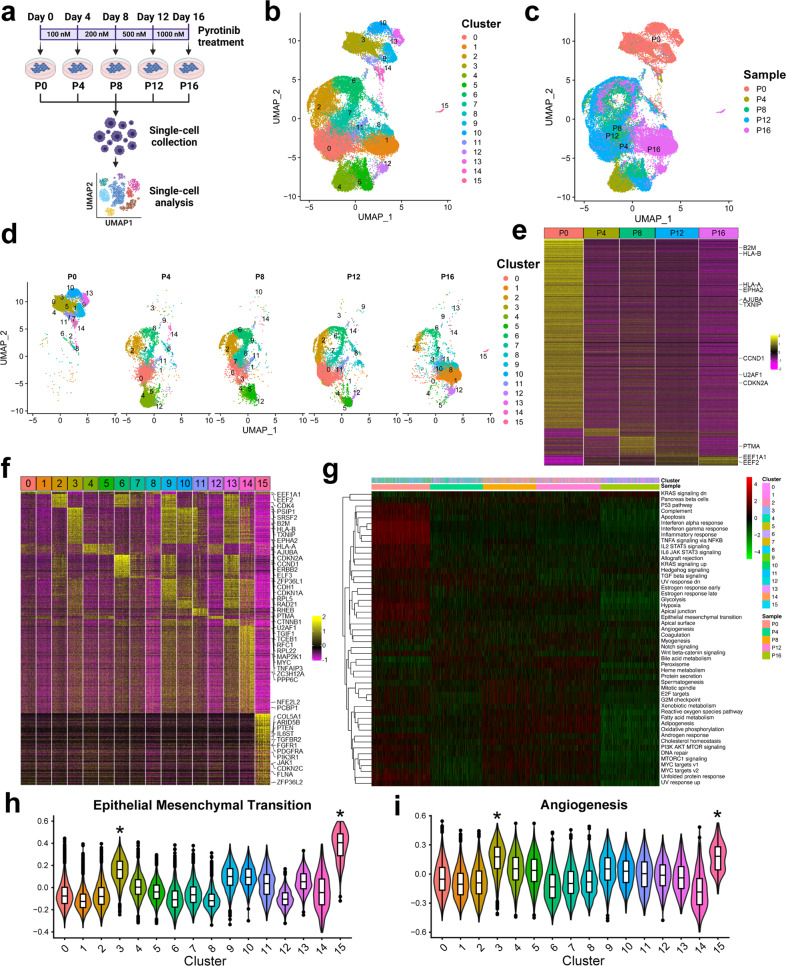
Single-cell RNA sequencing revealed transcriptomic evolution of NSCLC cells in response to pyrotinib. **a** Flow chart of pyrotinib treatment schedule and single-cell RNA sequencing procedures. Created with BioRender.com. **b** Umap illustration of cells colored by clusters. **c** Umap illustration of cells colored by samples. **d** Umap illustration of cells colored by clusters in separate samples. **e** Heatmap of significantly differentially expressed genes among the cells in various samples with cancer gene annotations. **f** Heatmap of significantly differentially expressed genes among the cells in various clusters with cancer gene annotations. 125 cells in each cluster were randomly chosen for illustration. **g** Heatmap illustrating the overview of GSVA scores of cancer hallmark signatures in different samples and clusters. **h**, **i** Violin plots of GSVA scores of (**h**) EMT and (**i**) angiogenesis for cells in different clusters. Significance * indicates FDR < 0.05 and |log2 fold change | > 0.5 compared to other groups

To better understand the expression patterns of each sample and cluster, we performed differential expression analysis by MAST, finding 6–299 significantly differentially expressed genes (DEGs; false discovery rate [FDR] < 0.05 and |log2 fold change | >0.5) in each sample (Fig. 1e) and 5–768 DEGs from each cluster (Fig. 1f). The untreated Sample P0 possessed the most (299) DEGs, including some driver genes,^4^ like *EPHA2*, *AJUBA*, *CCND1*, and *CDKN2A* (Supplementary Fig. S3a-d). Also, a total of 47 cancer driver genes (e.g., *CDK4*, *PTEN*, *MYC*, *PDGFRA*, and *CTNNB1*) were identified in the DEGs of various clusters (Supplementary Fig. S3f–j).^4^ Low expression levels of HER2 were reported in the resistance to other anti-HER2 therapies.^5^ Consistently, a transient elevation of *ERBB2* expression which encodes HER2 was detected in Sample P4 and its main cluster, Cluster 4, but it decreased in the following treatments (Supplementary Fig. S3e & k). Meanwhile, another target of pyrotinib, *EGFR*, remained relatively stable across samples. Then, the expression of the top 10 genes upregulated in each sample and top 5 genes enriched in each cluster were selected to show (Supplementary Fig. S4a, b). Interestingly, the isolated subpopulation Cluster 15 was featured by high expression of *MGP*, *COL1A1*, *COL1A2*, *SPARC*, and *COL3A1*. We found that single-cell differential expression analysis could distinguish more markers of subpopulations by its higher resolution.

Based on the expression profiles, we calculated 50 cancer hallmark scores of each cell by single-sample Gene Set Variation Analysis (ssGSVA) (Fig. 1g). Also, the average signature scores for every sample and cluster were calculated (Supplementary Fig. S5a, b) and compared using Wilcox test. The following GSVA signatures were enriched in Sample P0: interferon alpha response, interferon gamma response, TNFA signaling via NF-κB, inflammatory response, EMT, and hypoxia (Supplementary Fig. S5c–h). Besides, more pathways were identified in P0-related clusters (Cluster 3, 9, 10, 13, 14), like G2M checkpoint, Myc targets V2, mitotic spindle, unfolded protein response, and MTORC1 signaling. Additionally, the following pathways were enriched in the cells at transitional states: E2F targets, G2M checkpoint, interferon alpha response, and interferon gamma response. As reported by other studies, cell cycle regulation might contribute to the resistance to some anti-HER2 therapies. We found that a marker of G1-S cell cycle transition, *CDK4* expression was enriched in Cluster 2 which increased during the pyrotinib treatment in our study. Also, G2M checkpoint pathway scores were remarkably high in early-stage Clusters 2, 6, 9, and 13. These results together suggest the critical role of cell cycle regulation in pyrotinib resistance, indicating drugs targeted at cell cycles, like CDK4/6 inhibitors, might reverse the resistance. Moreover, the canonical malignant pathways, EMT (Fig. 1h) and angiogenesis (Fig. 1i), were significantly enriched in the isolated Cluster 15, suggesting this subpopulation is indispensable for surviving the pyrotinib treatment. Also, anti-angiogenesis regimens may overcome the resistance.

Furthermore, several immune-related signatures were found aberrantly regulated, indicating the potential association between immunotherapy outcomes and pyrotinib treatment. Thus, we explored the alterations of 5 immune checkpoints (*CD276*, *VTCN1*, *PDCD1LG2*, *CD274*, *LGALS9*) expressed on cancer cells at different stages of pyrotinib treatment, revealing that *CD274* (the gene encoding PD-L1) and *LGALS9* significantly decreased after pyrotinib treatment (Supplementary Fig. S6). These results suggest that pyrotinib may mediate outcomes of immunotherapy.

This is the first research investigating how NSCLC cells respond to pyrotinib treatment at the single-cell level. We sequenced ~8000 cells from each sample and included a total of 41804 cells after quality control for further analysis. To the best of our knowledge, our study includes more cells for subsequent analysis than other similar investigations using scRNA-seq. With the large number of single cells, we identified a small but critical subpopulation featured by upregulated EMT and angiogenesis pathways. Nevertheless, our study still has some limitations. Functional studies are needed to understand the mechanistic role of the identified resistant genes and pathways. Also, further validation of our immune-related findings needs more research involving animals or patient biopsies in the context of tumor microenvironment.

In conclusion, our analysis for the first time reveals the transcriptomic evolution of NSCLC cells in response to pytotinib administration using single-cell sequencing. We have identified important persistent subpopulations with aberrantly regulated genes and pathways, providing novel insights into the potential mechanisms of pyrotinib resistance and development of biomarkers for pyrotinib responses in NSCLC.

## Supplementary information

Supplementary Materials

## Data Availability

The datasets used and/or analyzed during the current study are available from the corresponding author on reasonable request.
