# Supplementary material for: Single-cell analysis reveals the potential mechanisms of pyrotinib resistance in non-small cell lung cancer
Source: Signal Transduct Target Ther. 2023 Jan 13;8:17. doi: 10.1038/s41392-022-01226-1 (PMC9837066; doi:10.1038/s41392-022-01226-1)
Supplement: Supplementary file 1 — Supplementary Materials [file 41392_2022_1226_MOESM1_ESM.docx]

Supplementary Materials for

Single-cell analysis reveals the potential mechanisms of pyrotinib resistance in non-small cell lung cancer

Xinfeng Wang, Yuan Li, Runsen Jin, Sufei Zheng, Yuejun Luo, Peng Wu, Zhanyu Wang, Yuxin Yao, Nan Sun*, Jie He*.

Correspondence to: Dr. Jie He (prof.jiehe@gmail.com) and Dr. Nan Sun (sunnan@vip.126.com)

**This PDF file includes:**

Materials and Methods

Figures. S1 to S6

Materials and Methods

Cell culture and treatment

The human bronchial epithelium cell line BEAS-2B and NSCLC cell lines was purchased from American Type Culture Collection (Manassas, VA). Human NSCLC cells were cultured in Roswell Park Memorial Institute (RPMI) 1640 media (Corning, Cat.#: 10-041-CV) containing 10% fetal calf serum (FBS; Gibco, Cat.#: 10100147), 100U/mL penicillin, 100mg/mL streptomycin (Gibco, Cat.#: 15140122), 1mM sodium pyruvate (Gibco, Cat.#: 11360070), and MEM Non-Essential Amino Acids (Gibco, Cat.#: 11140050). BEAS-2B cells were cultured in bronchial epithelial cell growth medium (BEGM) media (Lonza, Cat.#: CC-3170) with 10% FBS. All the cell lines were maintained under the normal culture condition (37℃ and 5% CO_2_). The short tandem repeat (STR) profile of the cell line was regularly detected to confirm its identity.

Cells were treated with pyrotinib at the increased doses of 100nM, 200nM, 500nM, and 1000nM, respectively. The drug pyrotinib was obtained from MedChemExpress (MCE, Cat.#: HY-104065) and dissolved to indicated concentrations by dimethyl sulfoxide (DMSO; Sigma, Cat.#: D2650).

Western blot

Protein extraction of cells and western blots were conducted according to the previous study ^1^. The primary antibodies were used at a dilution of 1:1000 including: anti-HER2 (Cat.#: 4290) and anti-GAPDH (Cat.#: 5174) from Cell Signaling Technology (CST) (Boston, MA). The used secondary antibody was anti-rabbit (Cat.#: A6154) from CST.

Single-cell RNA sequencing

At indicated time points, treated H358 cells were digested into single cells using 0.25% Trypsin-EDTA (Gibco, Cat.#: 25200056). Then, single-cell suspensions (300-600 living cells/mL) were loaded to Chromium single cell controller (10x Genomics) to generate single-cell gel beads by Chromium Single Cell B Chip Kit (10x Genomics, Cat.#: 1000074) and single cell 3 'Library and Gel Bead Kit V2 (10x Genomics, Cat.#: 1000075) following the manufacturer’s instructions. Subsequently, single cell RNA sequencing libraries were constructed, in which the captured single cells were lysed and the released RNA was barcoded by reverse transcription individually performed on a Thermal Cycler (Bio-Rad, Cat.#: S1000TM). Finally, the single-cell libraries were sequenced on an Illumina Novaseq6000 sequencer with a depth of ~100,000 reads per cell with pair-end 150 bp (PE150) reading strategy (performed by CapitalBio Technology, Beijing), and then mapped to the human genome (build hg19) by Cell Ranger (10x Genomics).

Data processing

ScRNA-seq data processing was completed using the Cell Ranger (3.1.0) pipeline. The hg19 reference dataset required for Cell Ranger analysis was obtained from 10x genomics. Briefly, first, the sequenced FASTQ files of 5 samples were aligned to the hg19 human reference genome by the STAR software using the Cell Ranger ‘*count*’ module. Second, five feature-barcode matrices were generated independently. Third, the five matrices were merged into an integrated object using *IntegrateData* function of Seurat package (version 4.0.2).

Identification of differentially expressed genes and marker genes

The cells which expressed gene number was less than 200 and the genes which were expressed less than 2 cells were regarded as abnormal and filtered out. Dimension reduction was performed using Principal Component Analysis (PCA), and visualization was realized by Uniform Manifold Approximation and Projection (UMAP) in Seurat. Then, the marker genes of each sample were identified by the Seurat *FindMakers* function. Besides, the differentially expressed genes (DEGs) were obtained by comparing cells from a specific sample to all the cells from the other 4 samples. Additionally, in order to identify the different expressed genes (DEGs) and marker genes under the same condition, we select the results showed by the UMAP method as the cell clusters in Seurat. For these 16 cells clusters, the specific marker genes of cells clusters were determined by the *FindMakers* function using MAST method ^2^. Specifically, the DEGs were collected by comparing cells from that cluster to all other cells of the rest of 15 clusters. Then, the genes with false discovery rate [FDR] < 0.05 and |log2 fold change| > 0.5 were selected as DEGs. Among these DEGs, genes that were identified as cancer drivers by Bailey et al.^3^ were indicated in heatmaps. Then, differentially expressed cancer driver genes associated with drug resistance according to literature review were selected for further illustration. Meanwhile, top 10 genes with the highest mean expression in each sample and top 5 genes in each cluster were respectively defined as marker genes.

Gene Set Variation Analysis (GSVA)

We performed single-sample Gene Set Variation Analysis (ssGSVA) to independently calculate the gene signature enrichment scores of individual single cells ^4^. The normalized gene expression matrix of all the single cells generated by ‘Seurat’ package was used to for analysis. Then, a gene set of 50 cancer hallmark signatures ^5^ was applied to the subsequent unbiased analysis. The consequent signature scores of each single cell were added 1 to get positive results, which were used for comparisons across various samples or clusters by the *FindMakers* function using Wilcox test based on the R package ‘Seurat’. Differentially enriched signatures of a sample or a cluster were considered as signatures with positive log2 fold change and adjusted FDR < 0.05 compared to the other samples or clusters, respectively.

Statistical analysis

Data processing of raw scRNA-seq data were performed using Cell Ranger (3.1.0) pipeline. All the following statistical analyses and graphs were generated using R software version 4.0.5. For descriptive analyses of cell numbers in different samples and clusters, ‘ggplot2’ package was used and ‘ggalluvial’ package was used to generate the Sankey diagram. The R package ‘Seurat’ was used for data normalization, sample quality control, dimension reduction, identification of DEGs and enriched signatures, and data visualization. The R package ‘gsva’ was applied to generating unbiased enrichment scores of individual single cells. For DEG identification, MAST method ^2^ was employed and the genes with false discovery rate [FDR] < 0.05 and |log2 fold change| > 0.5 compared to all the cells from other samples or clusters were considered as DEGs. For GSVA score comparison, Wilcox test was used and the gene signatures with positive log2 fold change and adjusted FDR < 0.05 were identified as enriched signatures.

**References**

1 Jin, R. *et al.* Desmoglein-2 modulates tumor progression and osimertinib drug resistance through the EGFR/Src/PAK1 pathway in lung adenocarcinoma. *Cancer Lett.* **483**, 46-58, (2020).

2 Finak, G. *et al.* MAST: a flexible statistical framework for assessing transcriptional changes and characterizing heterogeneity in single-cell RNA sequencing data. *Genome Biol.* **16**, 278, (2015).

3 Bailey, M. H. *et al.* Comprehensive Characterization of Cancer Driver Genes and Mutations. *Cell.* **173**, 371-385 e318, (2018).

4 Hänzelmann, S., Castelo, R. & Guinney, J. GSVA: gene set variation analysis for microarray and RNA-seq data. *BMC Bioinform.* **14**, 7, (2013).

5 Liberzon, A. *et al.* The Molecular Signatures Database (MSigDB) hallmark gene set collection. *Cell Syst.* **1**, 417-425, (2015).


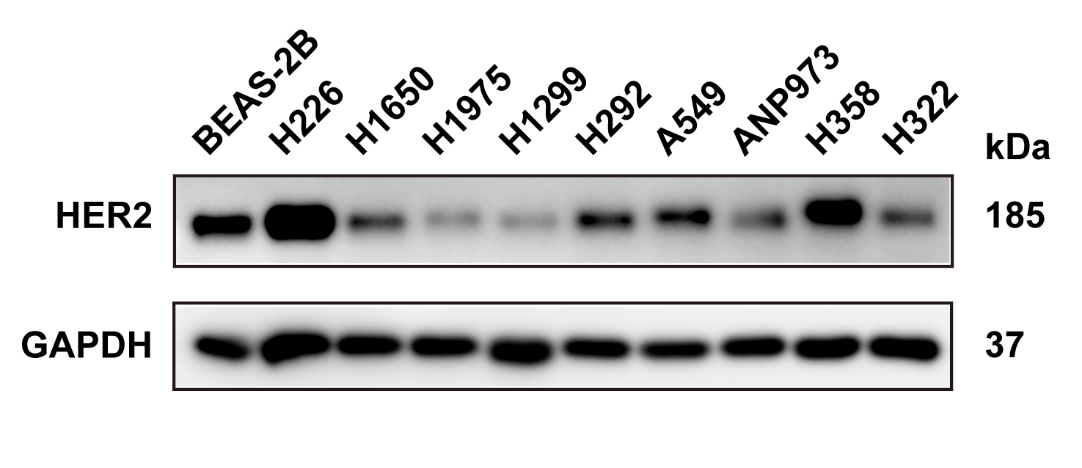
Figure. S1.

Expression of HER2 in human bronchial epithelium cell line BEAS-2B and nine NSCLC cell lines, GAPDH as the loading control.


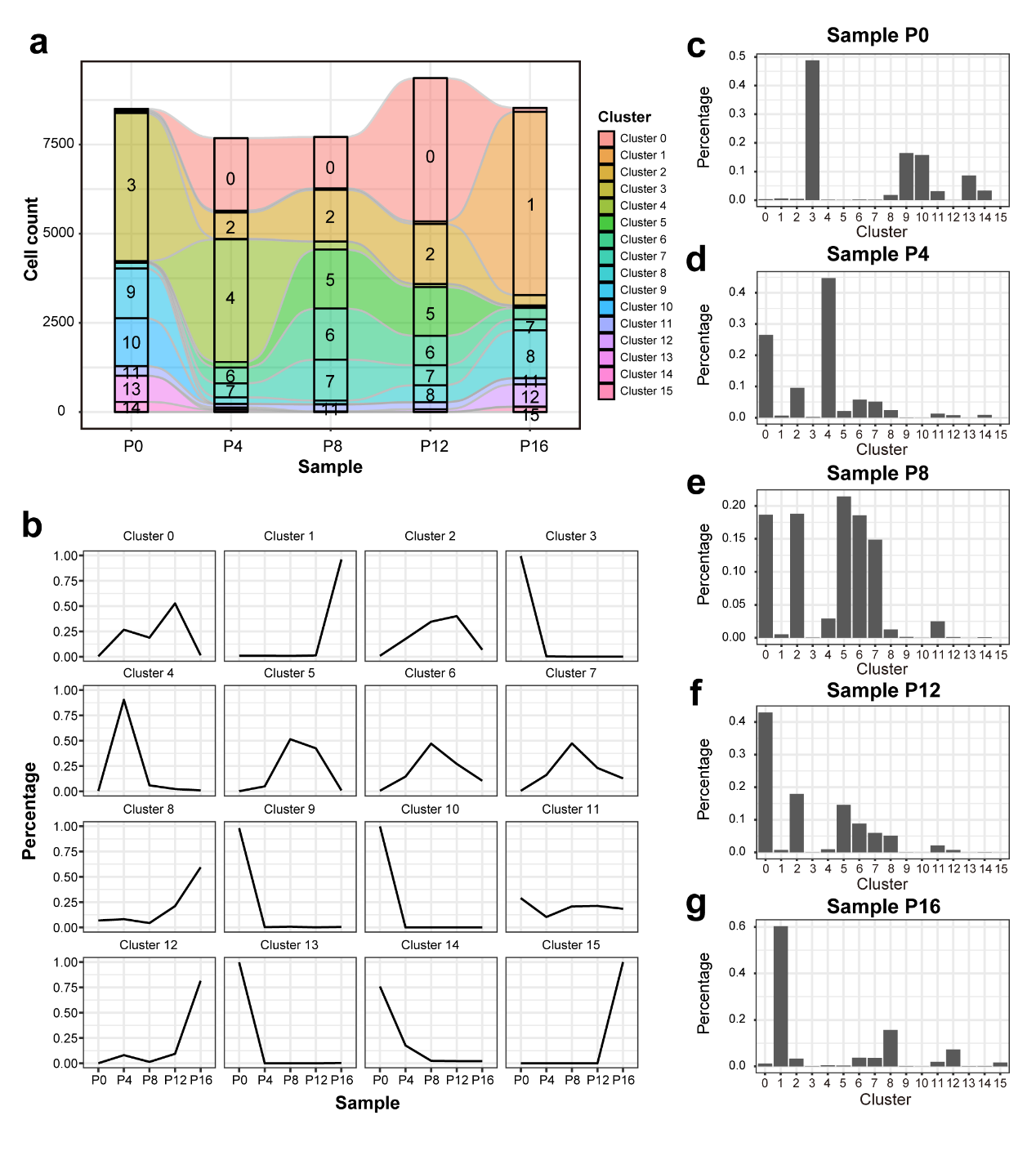
Figure. S2.

Distribution of cell numbers in different clusters or samples.

(a) Sankey plot showing the mapping relationship of cells between different samples and clusters. Names of major clusters in each sample are annotated in the column.

(b) Proportions of cells of different samples in various clusters.

(c-g) Distribution of cell numbers of various clusters in (c) Sample P0, (d) P4, (e) P8, (f) P12, and (g) P16.


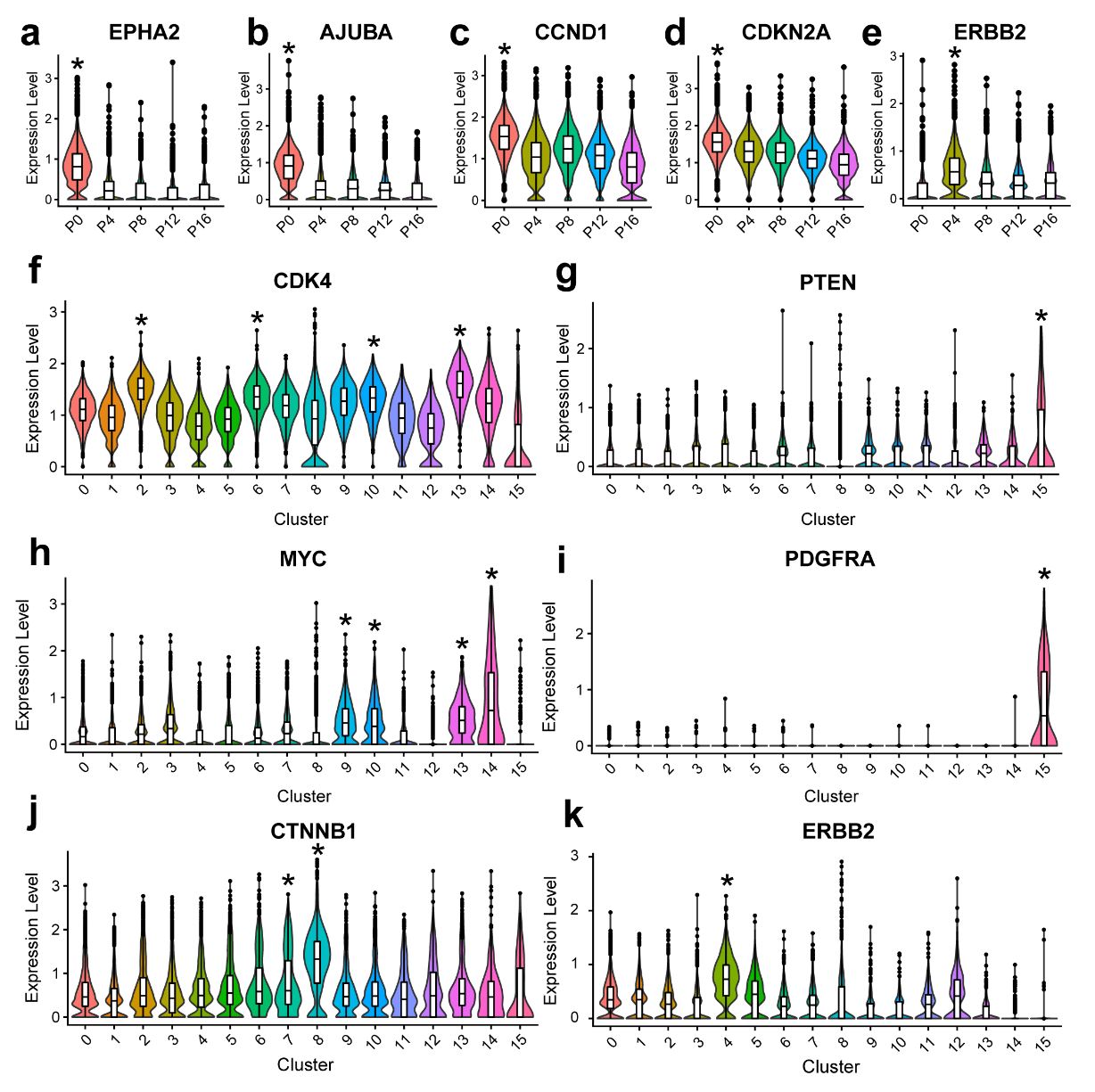


Figure. S3.

Single-cell RNA sequencing identified differentially expressed genes in various samples and clusters.

(a-e) Violin plots of single-cell RNA expression data of 5 cancer genes that were differentially expressed in samples.

(f-k) Violin plots of single-cell RNA expression data of 6 cancer genes that were differentially expressed in clusters.

Significance * indicates FDR < 0.05 and |log2 fold change| > 0.5 compared to other groups.


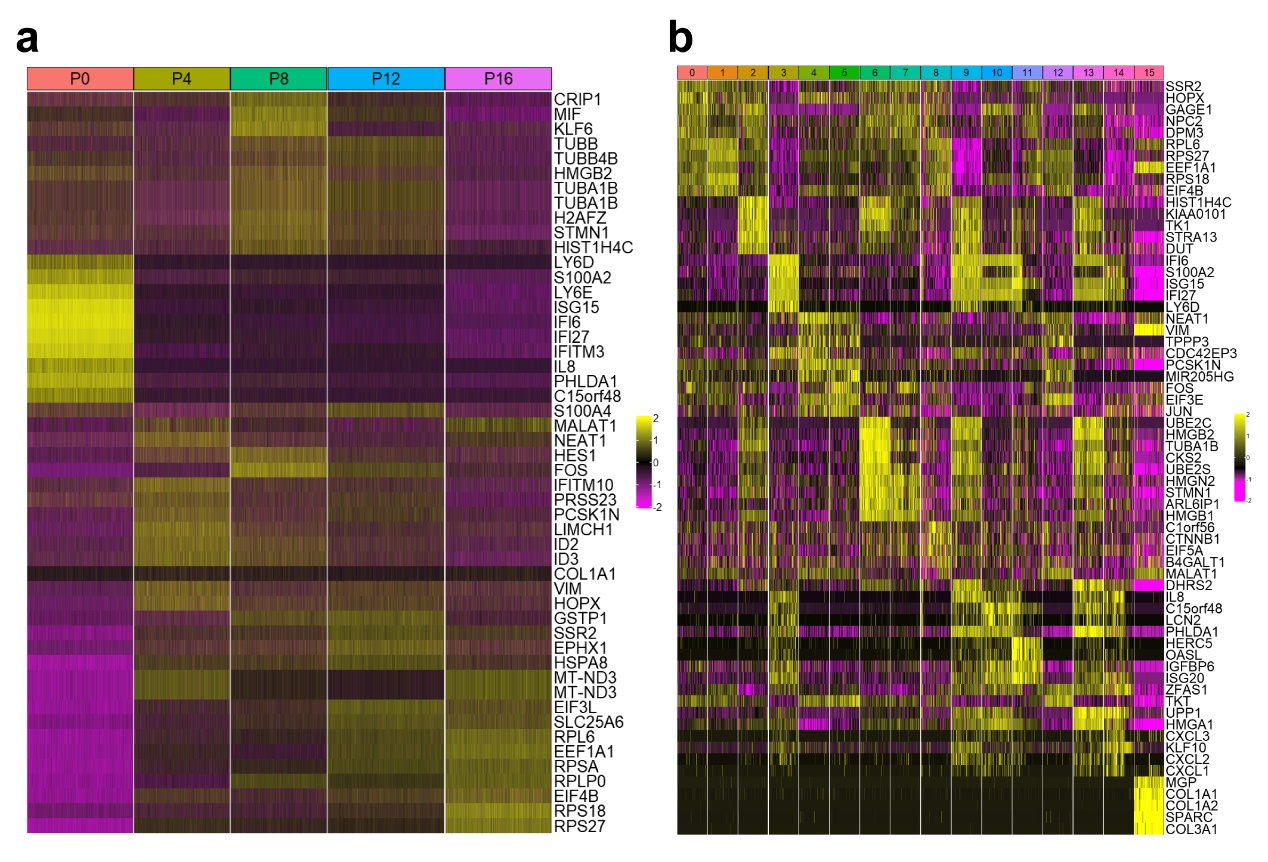
Figure. S4.

Single-cell RNA sequencing identified differentially expressed genes in various samples and clusters.

(a) Heatmap of top 10 significantly differentially expressed genes among the cells in various samples.

(b) Heatmap of top 5 significantly differentially expressed genes among the cells in various clusters. 125 cells in each cluster were randomly chosen for illustration.


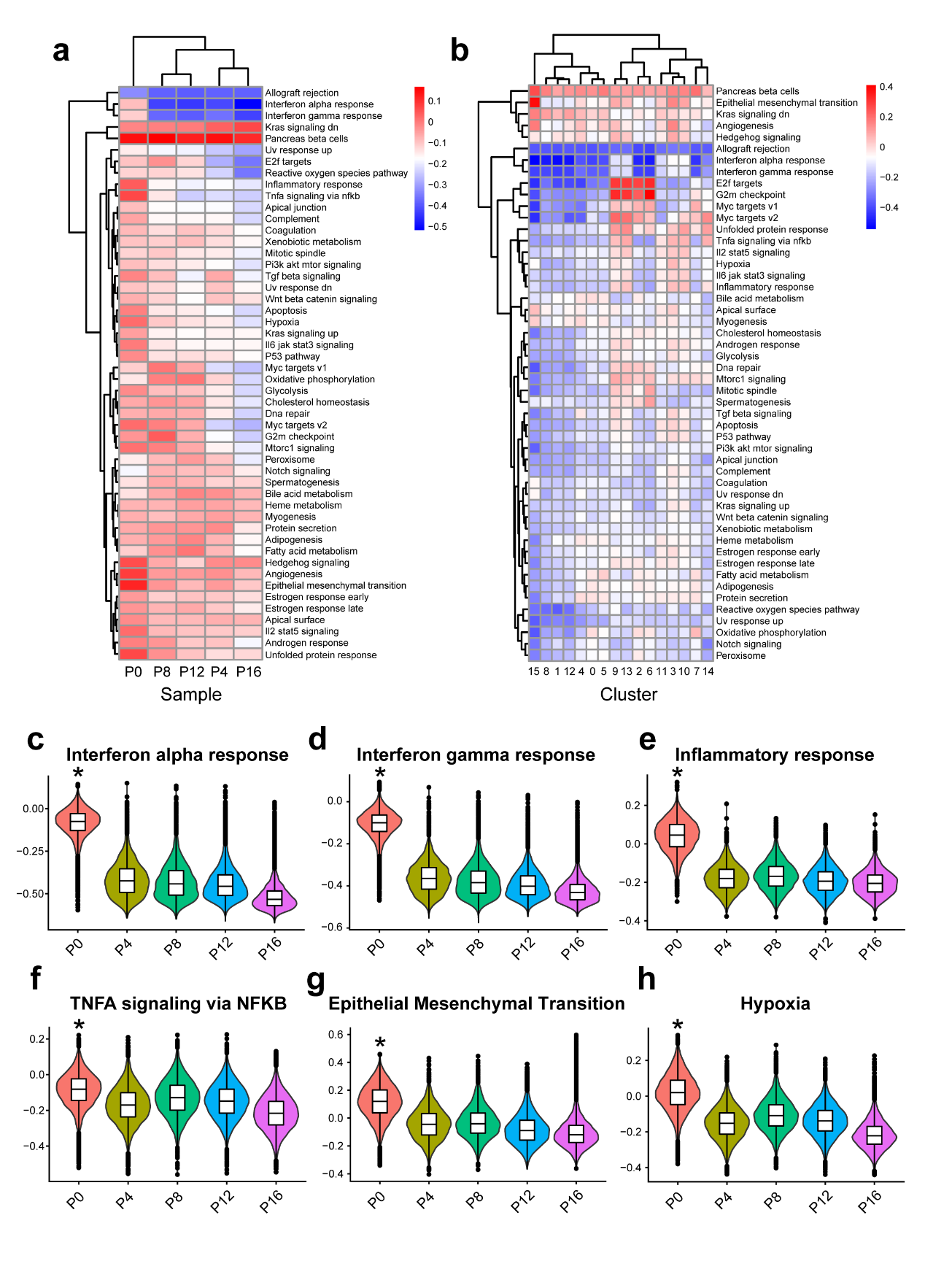
Figure. S5.

Single-cell RNA sequencing identified differentially regulated cancer hallmark pathways in various samples and clusters.

(a-b) Heatmaps illustrating the mean values of GSVA scores of cancer hallmark signatures in various samples (a) and clusters (b).

(c-h) Violin plots of 6 GSVA scores differentially upregulated in cells from Sample P0.

Significance * indicates FDR < 0.05 and |log2 fold change| > 0.5 compared to other groups.


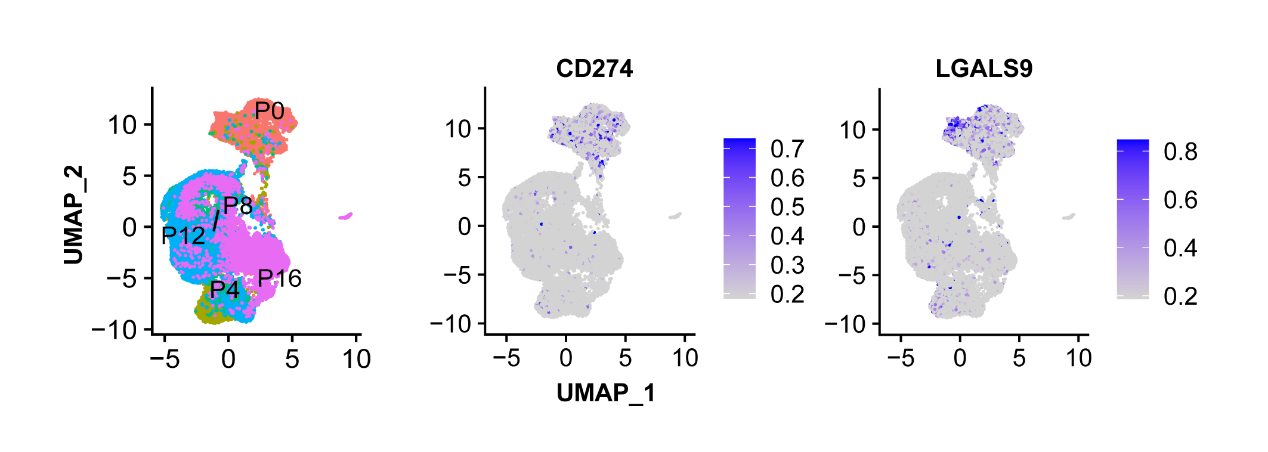
Figure. S6.

Expression of immune checkpoint genes, CD274 and LGALS9, downregulated in the treated samples.
